#  Letter regarding “The usefulness of the genetic panel in the classification and refinement of diagnostic accuracy of Mexican patients with Marfan syndrome and other connective tissue disorders”

**DOI:** 10.17305/bb.2024.10799

**Published:** 2024-10-01

**Authors:** Ana M Serrano-Ardila, Gilda S Garza-Mayén, Alejandro Gaviño-Vergara

**Affiliations:** 1 The American British Cowdray Medical Center, Mexico City, Mexico; 2 Unidad de Genética, Hospital Ángeles Lomas, Mexico City, Mexico; 3 Clinical Genetics CRIT Quintana Roo, Mexico

Dear Editor,

We have read the article “The usefulness of the genetic panel in the classification and refinement of diagnostic accuracy of Mexican patients with Marfan syndrome and other connective tissue disorders”, recently published in your esteemed journal [1].

We are a team dedicated to diagnosing, approaching, and managing patients with connective tissue disorders, particularly hypermobile spectrum disorders (HSD) and Ehlers–Danlos syndromes (EDS). We appreciate the research group’s effort to address the complexity of connective tissue disorders using a multi-panel genetic approach and their analysis of genotype–phenotype associations in a cohort of Mexican patients.

However, we would like to express our concern regarding two specific points that we consider crucial for the comprehensive understanding and management of these disorders.

First, we must question why the study did not involve clinical geneticists in analyzing and discussing the results. Including genetic specialists could have enriched the approach and interpretation of the findings, given that the genomics of connective tissue disorders is a complex field requiring specialized knowledge for accurate and thorough evaluation. A clinical geneticist’s participation in the clinical assessment of these patients is essential since performing a detailed physical examination allows us to classify the patients better and make the phenotype and genotype classification.

Even though there are no clear phenotype–genotype correlations in this study, molecular studies in connective tissue disorders have predominantly served worldwide to distinguish between differential diagnoses due to the broad clinical spectrum and variable expressivity in these diseases, as indicated in the same article. Therefore, another limitation of the study analysis is that a panel of 174 genes might fall short of making comprehensive conclusions about connective tissue diseases, considering there are currently 545 gene associations with joint hypermobility [2].

Additionally, we consider that conclusions on phenotype–genotype correlations require more knowledge of variant classification since variants, such as nonsense, frameshift, canonical ± 1 or 2 splice sites, initiation codon, and single or multiple exon deletions are part of the “null variants.” Furthermore, the impact of missense variants depends on criteria, such as the evolutionary conservation of an amino acid or nucleotide, the location and context within the protein sequence, and the biochemical consequence, as widely known since 2015 [3].

Second, we noticed that the study’s approach did not mention hypermobile spectrum disorders and EDS. Considering these diagnoses’ prevalence and clinical relevance, this omission is significant. Although not rare, hypermobile spectrum disorders and EDS are rarely diagnosed, highlighting the need to include them in studies aiming to improve the diagnostic accuracy of connective tissue disorders. Ignoring these diagnoses can lead to underestimations in prevalence and incomplete therapeutic approaches [4].

We hope these comments are helpful for future research and advocate for a more inclusive approach that considers the participation of geneticists and the inclusion of diagnoses, such as hypermobile spectrum disorders and EDS.

We appreciate the attention to these observations and look forward to a response.
